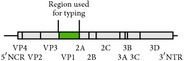# Direct Detection and Identification of Enteroviruses from Faeces of Healthy Nigerian Children Using a Cell-Culture Independent RT-Seminested PCR Assay

**DOI:** 10.1155/2016/1412838

**Published:** 2016-03-20

**Authors:** Temitope Oluwasegun Cephas Faleye, Moses Olubusuyi Adewumi, Bamidele Atinuke Coker, Felix Yasha Nudamajo, Johnson Adekunle Adeniji

**Affiliations:** ^1^Department of Virology, College of Medicine, University of Ibadan, Ibadan, Oyo State, Nigeria; ^2^Department of Microbiology, Faculty of Science, Ekiti State University, Ado Ekiti, Ekiti, Nigeria; ^3^Department of Microbiology, Faculty of Science, University of Ibadan, Ibadan, Oyo State, Nigeria; ^4^WHO National Polio Laboratory, University of Ibadan, Ibadan, Oyo State, Nigeria

## Abstract

Recently, a cell-culture independent protocol for detection of enteroviruses from clinical specimen was recommended by the WHO for surveillance alongside the previously established protocols. Here, we investigated whether this new protocol will show the same enterovirus diversity landscape as the established cell-culture dependent protocols. Faecal samples were collected from sixty apparently healthy children in Ibadan, Nigeria. Samples were resuspended in phosphate buffered saline, RNA was extracted, and the VP1 gene was amplified using WHO recommended RT-snPCR protocol. Amplicons were sequenced and sequences subjected to phylogenetic analysis. Fifteen (25%) of the 60 samples yielded the expected band size. Of the 15 amplicons sequenced, 12 were exploitable. The remaining 3 had electropherograms with multiple peaks and were unexploitable. Eleven of the 12 exploitable sequences were identified as Coxsackievirus A1 (CVA1), CVA3, CVA4, CVA8, CVA20, echovirus 32 (E32), enterovirus 71 (EV71), EVB80, and EVC99. Subsequently, the last exploitable sequence was identified as enterobacteriophage baseplate gene by nucleotide BLAST. The results of this study document the first description of molecular sequence data on CVA1, CVA8, and E32 strains present in Nigeria. The result further showed that species A enteroviruses were more commonly detected in the region when cell-culture bias is bypassed.

## 1. Introduction

Enterovirus infections have been associated with an array of clinical manifestations that range from aseptic meningitis through type 1 diabetes to acute flaccid paralysis (AFP) among others [1]. However, these clinically manifest infections represent <10% of the actual burden of enterovirus infections and have been estimated to amount to about 10–15 million cases annually in the United States alone [2]. The remaining over 90% of such infections are asymptomatic [3].

Enteroviruses are nonenveloped viruses with a diameter of 20–30 nM. Within the virion is a positive sense, single stranded RNA genome that is approximately 7,500 nt long. The genome has one open reading frame (ORF), the polyprotein product of which is autocatalytically cleaved into structural (VP1–VP4) and nonstructural (2A–3D) proteins. The ORF is flanked on both ends by untranslated regions (UTRs) and a poly-A tail at the 3′-end.

Enteroviruses belong to the genus* Enterovirus* in the family Picornaviridae, order Picornavirales. Classification of enteroviruses used to be based on virion particle structure, tissue culture growth properties, and pathogenesis in humans and animals [4]. However, classification is now based on virus genomics [4] and most especially phylogeny of the VP1 protein [5–16]. Based on the recent classification (http://www.picornaviridae.com/), there are 12 species in the genus, four (*Enterovirus* species A–species D [EVA-EVD]) of which were previously known as “human enteroviruses.” At the time of writing, EVA contained 25 serotypes made up of 11 CVAs, 10 numbered enteroviruses, and four (4) enteroviruses isolated from nonhuman primates. EVB contained 63 serotypes consisting of one (1) CVA, six (6) CVBs, 28 echoviruses, 27 numbered enteroviruses, and one (1) enterovirus isolated from a nonhuman primate. EVC contained 23 serotypes consisting of nine (9) CVAs, three (3) poliovirus serotypes, and eleven (11) numbered enteroviruses. EVD contained five (5) serotypes consisting only of numbered enteroviruses (http://www.picornaviridae.com/).

Besides the fact that EVB has the highest number of serotypes, it is also the most commonly detected [15–21]. It has however been suggested that this phenomenon (called the EVB bias) might be an artefact of the strategy used for enterovirus isolation and might not be truly representative of the enterovirus diversity landscape [21, 22].

Almost all previous studies documenting enterovirus diversity in Nigeria [15, 16, 23, 24] clearly showed the preponderance of EVB. However, all such studies have been cell-culture based and mainly used the RD cell line which has been suggested to be the EVB bias [15–21], for enterovirus isolation. The only study that did differently [22] used MCF 7 and LLC-MK2 cell lines for enterovirus isolation and documented an increase in the detection rate of enterovirus species C (EVC) members.

Recently, Nix et al.'s [25] cell-culture independent protocol for direct detection of enteroviruses from clinical specimen was recommended [4] for enterovirus surveillance alongside the previously established protocols [26, 27]. In this study, we investigated whether this strategy will show the same enterovirus diversity landscape as the established cell-culture dependent protocols [26, 27] and document a preponderance of EVAs in Southwestern Nigeria.

## 2. Methodology

### 2.1. Sample Collection and Storage

Faecal samples were collected from sixty (male = 37, female = 23) apparently healthy children aged 1 to 10 years attending public primary schools in Ibadan, Nigeria. Samples were collected from the pupils after approval and consent were secured from the school administration and the guardian or parents of the children, respectively. Stool samples were collected from each of the children into appropriately labelled sterile collection bottles. Samples were then transported to the laboratory in the Department of Virology, College of Medicine, University College Hospital, Ibadan, Nigeria, in a cooler filled with ice packs to maintain a temperature of about 4°C. On arrival at the laboratory, the stool specimens were stored at −20°C until analysis.

### 2.2. Sample Processing

About one gram of each stool specimen was diluted in 3 mL phosphate buffered saline (PBS), 1 mL chloroform, and one gram of glass beads. The mixture was then vortexed for 20 minutes and thereafter centrifuged at 3000 rpm for 20 minutes. Subsequently, 2 mL of the supernatant was aliquoted in 1 mL volumes into cryovials. One vial was stored at −20°C while the other was analysed further.

### 2.3. RNA Extraction and cDNA Synthesis

JenaBioscience RNA extraction kit (Jena Bioscience, Jena, Germany) was used for viral RNA extraction according to the manufacturer's instructions. Script cDNA synthesis kit (Jena Bioscience, Jena, Germany) was used for cDNA synthesis according to the manufacturer's instructions. However, instead of random hexamers, primers AN32, AN33, AN34, and AN35 [25] were used for cDNA synthesis.

### 2.4. Enterovirus VP1 Gene Seminested PCR (snPCR) Assay

Primers were made in 25 *μ*M concentrations and PCR was done in 30 *μ*L reactions. The first-round PCR contained 2 *μ*L of each of primers 224 and 222 (Nix et al., 2006), 6 *μ*L of Red Load Taq, 10 *μ*L of cDNA, and 10 *μ*L of RNase-free water. Thermal cycling was done in a Veriti thermal cycler (Applied Biosystems, California, USA). Thermal cycling conditions were 94°C for 3 minutes followed by 45 cycles at 94°C for 30 seconds, 42°C for 30 seconds, and 60°C for 60 seconds with ramp of 40% from 42°C to 60°C. This was then followed by 72°C for 7 minutes and held at 4°C till being terminated. The second-round PCR was carried out with the first-round PCR product as template, with similar thermal cycling conditions except for the extension time that was reduced to 30 seconds, and the primers were substituted with AN89 and AN88 [25], respectively. Subsequently, PCR products were resolved on 2% agarose gel stained with ethidium bromide and viewed using a UV transilluminator.

### 2.5. Nucleotide Sequencing

All amplicons were shipped to Macrogen Inc., Seoul, South Korea, for purification and sequencing of only the bands of the expected size. Primers AN88 and AN89 were used for sequencing. Afterwards, the enterovirus genotyping tool [28] was used for enterovirus species and genotype determination.

### 2.6. Phylogenetic Analysis

To align the sequences described in this study with reference sequences downloaded from the GenBank, the ClustalW program in the MEGA 5 software [29] was used with default settings. Afterwards, neighbour-joining trees were constructed with the Kimura-2 parameter model [30] and 1,000 bootstrap replicates using the same MEGA 5 software.

### 2.7. Nucleotide Sequence Accession Numbers

All the sequences reported in this study have been deposited in GenBank under accession numbers KT717062–KT717072.

## 3. Results

### 3.1. RT-snPCR Result

A total of 15 (25%) of the sixty (60) stool samples screened yielded the expected band size for the enterovirus VP1 gene detection RT-snPCR screen (Table 1). Of the 37 and 23 samples collected from the male and female participants, respectively, 11 (29.73%) and four (17.39%) yielded the expected band size (Table 1).

### 3.2. Virus Identification

Of the 15 amplicons subjected to sequencing, only 12 were exploitable. The remaining 3 were unexploitable due to the presence of multiple peaks in their electropherograms. Eleven (11) of the 12 exploitable sequences were successfully typed by the enterovirus genotyping tool (EGT) as Coxsackievirus A1 (CVA1) (1 strain), CVA3 (1 strain), CVA4 (1 strain), CVA8 (2 strains), CVA20 (1 strain), echovirus 32 (E32) (1 strain), Enterovirus A71 (EVA71) (2 strains), EVB80 (1 strain), and EVC99 (1 strain) (Table 1). Subsequently, the last exploitable sequence was subjected to a BLAST search and found to be most similar to an enterobacteriophage baseplate gene (Table 1). Based on the eleven (11) typed strains, enterovirus species A, B, C, and D accounted for 54.55%, 18.18%, 27.27%, and 0% of the detected strains.

### 3.3. Phylogenetic Analysis

**Figure 1:**
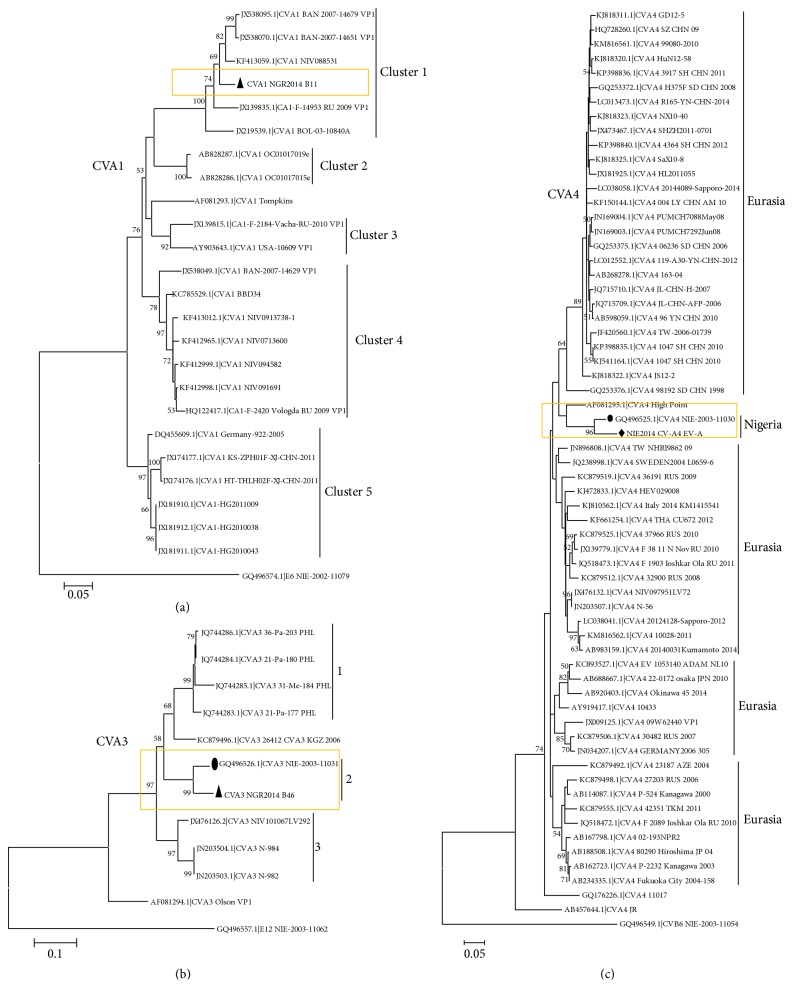
Phylogenetic relationship of recovered CVA1 (a), CVA3 (b), and CVA4 (c) strains. The phylogram is based on alignment of the partial VP1 sequences. The newly sequenced strains and previous strains from the region are highlighted with black triangles or diamonds and circles, respectively. The GenBank accession number of the strains is indicated in the phylogram. Bootstrap values are indicated if >50%.

With respect to CVA1, the sequences obtained from GenBank and the one described in this study clustered into five different groups with strong bootstrap support (Figure 1(a)). The CVA1 sequence of Nigerian origin described in this study clustered with sequences from Eurasia (Figure 1(a)). In the CVA3 phylogram, there are three distinct clusters with strong bootstrap support (Figure 1(b)). Within cluster 2, the Nigerian CVA3 detected in this study clustered with another CVA3 previously detected in Nigeria in 2003 [15] (Figure 1(b)). Just like for CVA3, the Nigerian CVA4 detected in this study clustered with another CVA4 previously detected in Nigeria in 2003 [15] (Figure 1(c)).

**Figure 2:**
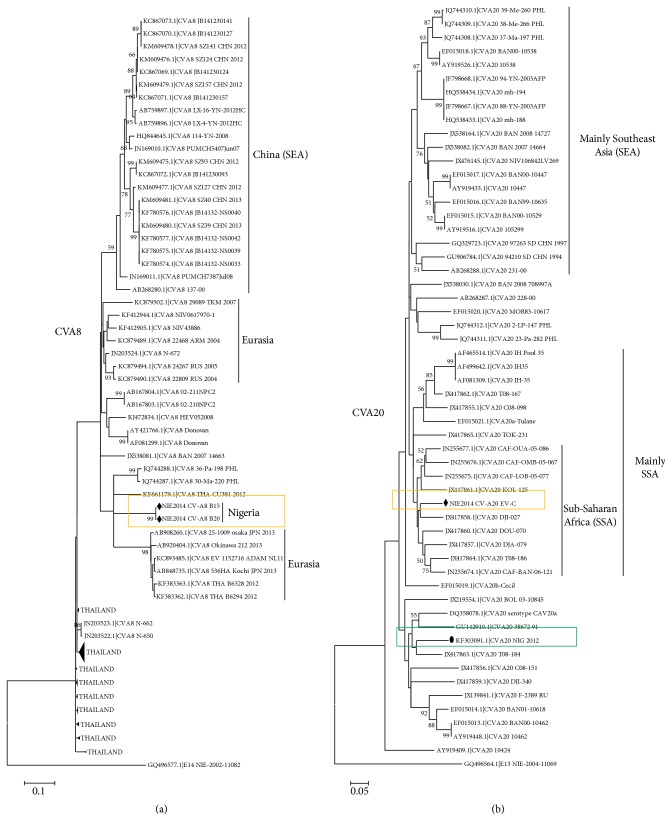
Phylogenetic relationship of recovered CVA8 (a) and CVA20 (b) strains. The phylogram is based on alignment of the partial VP1 sequences. The newly sequenced strains and previous strains from the region are highlighted with black triangles or diamonds and circles, respectively. The GenBank accession number of the strains is indicated in the phylogram. Bootstrap values are indicated if >50%.

The two CVA8 sequences described in this study clustered with one another, with strong bootstrap support. These CVA8 sequences did not appear to be too closely related to any of the CVA8 sequences in the phylogram (Figure 2(a)). The CVA20 sequence described in this study, on the other hand, did not cluster with that previously detected in the region in 2012 (Figure 2(b)). Rather, it clustered with other CVA20 sequences recently described in Central African Republic [19] and Cameroon [21] (Figure 2(b)).

**Figure 3:**
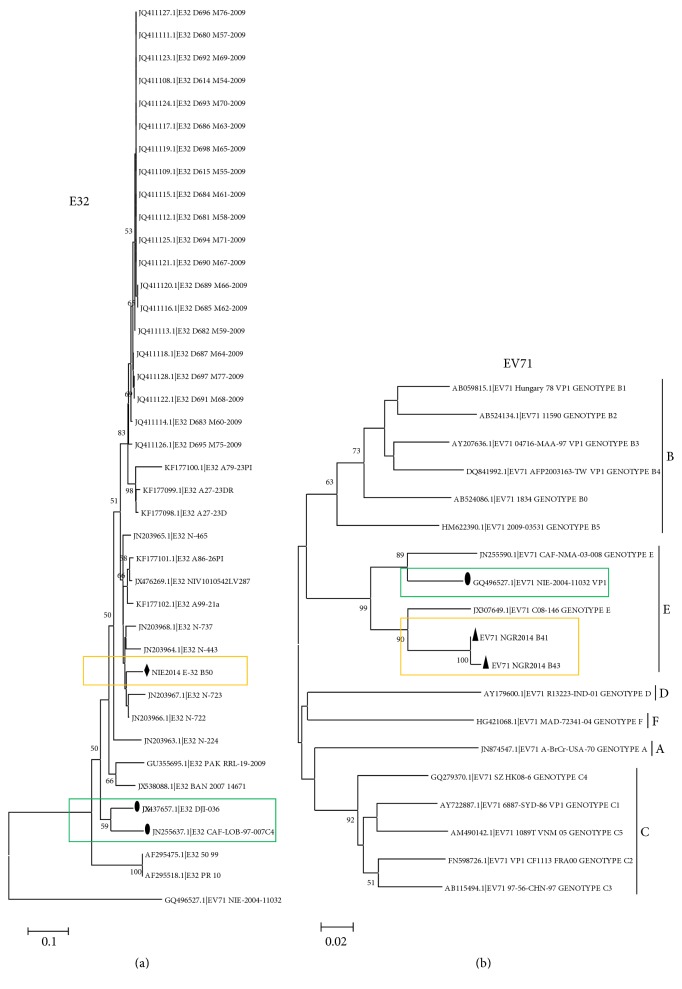
Phylogenetic relationship of recovered E32 (a) and EV71 (b) strains. The phylogram is based on alignment of the partial VP1 sequences. The newly sequenced strains and previous strains from the region are highlighted with black triangles or diamonds and circles, respectively. The GenBank accession number of the strains is indicated in the phylogram. Bootstrap values are indicated if >50%.

The E32 sequence described in this study did not cluster with other E32 sequences recently described in Central African Republic [19] and Cameroon [21] (Figure 3(a)). Rather, it clustered with E32 sequences recently described in India [31]. On the other hand, both EV71 sequences described in this study clustered together with strong bootstrap support in genotype E (Figure 3(b)). Though this genotype consisted only of sequences from sub-Saharan Africa [15, 19, 21], the EV71 sequences described in this study clustered with the EV71 from Cameroon [21] while the EV71 previously described in Nigeria in 2004 clustered with that from Central African Republic [19] (Figure 3(b)).

The single EVB80 sequence described in this study clustered, with strong bootstrap support, with others we recently found in 2014 in Nigerian children diagnosed with AFP (unpublished data). Contrary to the situation with EVB80, the EVC99 sequence described in this study was very different from the one we recently found in 2014 in Nigerian children diagnosed with AFP (unpublished data). Though they were both found in Nigeria in 2014, they appear to be most closely related to EVC99 sequences from Cameroon [21], but with different genotypes.

## 4. Discussion

### 4.1. Enterovirus Detection Rate

Considering that only eleven of the samples could be unequivocally shown to contain enteroviruses, the results of this study show enterovirus detection rate of 18.3% (11/60) in apparently healthy school aged children in Ibadan, Southwestern Nigeria. This is higher than the 5.5% and 10% described in previous studies from apparently healthy school aged children in Southwestern [15] and Northeastern [24] Nigeria, respectively. This might be a reflection of the impact of using different detection protocols. While direct detection of enterovirus genome from the clinical sample was used in this study, the other studies [15, 24] used a cell-culture based algorithm, particularly a combination of RD and L20b cell lines, as previously recommended by the WHO [27]. This might therefore suggest that the cell-culture independent protocol of Nix et al. [25] for direct detection of enteroviruses from clinical specimen might be more sensitive than the WHO cell-culture based protocol [27]. However, such conclusion cannot be reached unequivocally, because the samples analysed in this study were not simultaneously screened using both the RD and L20b cell line based and the cell-culture independent protocols.

### 4.2. Enterovirus Species Diversity Landscape

The results of this study showed that species A enteroviruses were more commonly detected (54.55%) than members of the other enterovirus species (Table 1). This contradicts the findings of previous studies from the region [15, 16, 23, 24] which gave the impression that species B enteroviruses were the most commonly circulating. The true meaning and significance of this contradiction is difficult to determine considering that the same samples were not subjected to cell culture using the protocols previously documented in the region. However, this finding might be better descriptive of the enterovirus diversity landscape in the region because it bypasses cell-culture bias.

It can however be argued that the picture of the enterovirus diversity landscape painted by this cell-culture independent assay may just be a reflection of the primer specificities. Consequently, the tilt in the landscape towards species A members might not be a true reflection of the diversity landscape. However, considering that, as opposed to species A, C, and D which all individually have less than 30 serotypes documented, species B has over 60 serotypes documented (http://www.picornaviridae.com/), more species B members would have been considered during the primer design process [7, 9, 25, 32]. As a result, the primers should be biased towards species B rather than other species. Hence, the preponderance of species A enterovirus members in this population is unlikely to be as a result of primer bias.

### 4.3. Enterovirus Serotypes Detected

The results of this study showed the presence of nine (9) different serotypes of nonpolio enteroviruses (CVA1, CVA3, CVA4, CVA8, CVA20, E32, EVA71, EVB80, and EVC99) in apparently healthy, school aged children in Ibadan, Southwestern Nigeria, in 2014 (Table 1). This study documents the first description of molecular sequence data on CVA1, CVA8, and E32 strains present in Nigeria. Though this is also the first publication of molecular sequence data of EVB80 and EVC99 from Nigeria, we had previously detected EVB80 and EVC99 in children with AFP in 2014 (unpublished data). Furthermore, we had previously described CVA20 in environmental samples in 2012 [33] and as for CVA3, CVA4, and EV71, molecular sequence data of strains circulating over ten (10) years ago were previously described [15].

### 4.4. Enterovirus Regional Confinement Hypothesis

The discovery of EV71 genotype E in Nigeria in 2004 [15, 23] and the subsequent detection of more members of the genotype in Central African Republic [19] and Cameroon [21] led to the hypothesis that certain enteroviruses strains circulating in sub-Saharan Africa might be confined to the region (the regional confinement hypothesis [RCH]). The recent discovery of EV71 genotype F in Madagascar [34] further supports the RCH. It was postulated that paucity of data on enterovirus genotypes circulating in the region may be responsible for the delayed discovery of these EV71 genotypes [19, 21]. However, subsequent to the discovery of EV71 genotype F, it has recently been shown that both genotypes might have diverged from their independent, most recent common ancestors in the 1990s [34].

**Figure 4:**
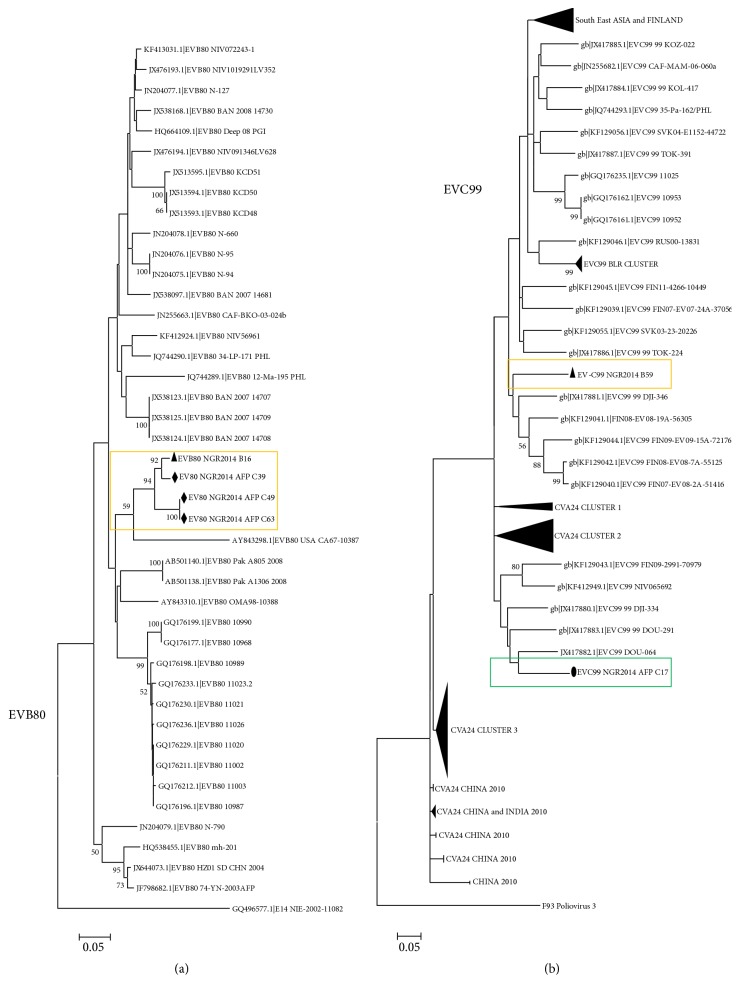
Phylogenetic relationship of recovered EVB80 (a) and EVC99 (b) strains. The phylogram is based on alignment of the partial VP1 sequences. The newly sequenced strains and previous strains from the region are highlighted with black triangles and circles, respectively. The GenBank accession number of the strains is indicated in the phylogram. Bootstrap values are indicated if >50%.

The EV71 strains detected in this study belonged to genotype E (Figure 3(b)), further confirming the RCH. Furthermore, CVA3 (Figure 1(b)), CVA4 (Figure 1(c)), CVA20 (Figure 2(b)), and EVC99 (Figure 4(b)) also showed evidence in support of the RCH. However, though regionally confined, the actual EV71 clade recovered in Nigeria in 2004 appears to have been replaced by a new clade (Figure 3(b)). In similar light, though regionally confined, the EVC99 strain detected in this study is different from that we recently detected in a child diagnosed with AFP (unpublished data) (Figure 4(b)). This therefore suggests the simultaneous circulation of two distinct clades of EVC99 in the country. On the other hand, genotype replacement has been observed for CVA20 (Figure 2(b)). However, the same cannot be said for CVA3 and CVA4 due to paucity of molecular sequence data from the region on these genotypes. Though the isolates of CVA3 and CVA4 detected in this study are similar to those detected over 10 years ago from the same region (Figures 1(b) and 1(c)), characterizing more isolates from the intervening years will help better understand the evolutionary dynamics of these serotypes.

The E32 isolate described in this study appeared to be more closely related to isolates from southeast Asia than those from sub-Saharan Africa (Figure 3(a)). On the one hand, this calls to question the RCH. However, on the other hand, it brings to the fore another salient underdiscussed issue concerning enterovirus identification that gives newcomers to the field some headache. Sequences of the VP1 gene are usually used for enterovirus identification. However, while the most appropriate strategy would be to amplify the entire VP1 gene, most protocols amplify either the 5′- or the 3′-end. For the newbie, it can be quite confusing to find out whether the partial VP1 gene is from the 5′- or the 3′-end of the gene. However, the enterovirus genotyping tool [28] helps to resolve this by giving a graphic view of the physical location on any VP1 gene (complete or partial) submitted as query sequence, thereby helping to determine whether the sequence in question is the complete gene or 5′- or 3′-end of the gene (Tables 2(a) and 2(b)).

Nix et al.'s [25] protocol is an upgrade of Oberste et al.'s [7, 9] protocol and amplifies the 5′-end of the VP1 gene. Consequently, sequences generated using this protocol can only be compared to those generated using similar protocols that amplify the 5′-end or complete VP1 gene. Sequences generated from protocols that amplify the 3′-end of the VP1 gene like those of Oberste et al. [6], Casas et al. [10], and Caro et al. [12] or any iteration of these are of no value for phylogenetic analysis of VP1 gene sequences generated using Nix et al.'s [25] protocol. This is because it will be impossible to align partial VP1 sequences generated using Nix et al.'s [25] protocol with those from protocols that amplify the 3′-end of the gene.

There are a significant number of sequences of this sort in GenBank and some of the sub-Saharan enterovirus sequences fall into this category alongside those from other world regions (Tables 2(a) and 2(b)). This dichotomy undermines the capacity to better investigate the RCH with the data at hand and necessitates the need to have a second look at the adoption and use of Nix et al.'s [25] protocol and other protocols that do not amplify the entire VP1 gene for studies focused on investigating the RCH. This dichotomy accounts for why the regional confinement of strains of E32, as well as CVA1, CVA8, and EVB80, detected in this study could not be exhaustively determined despite the availability of sequence data from sub-Saharan Africa strains in the nucleotide databases. Therefore, other cell-culture independent protocols for direct detection of enteroviruses from clinical samples like the ECRA recently described by Arita et al. [35], which have the capacity to amplify the complete VP1 gene, should be further investigated and developed to tools that are affordable and field deployable, especially in resource limited settings.

### 4.5. What Happens with Coinfections?

Subsequent to the completion of this study, it was observed that, in cases of enterovirus coinfection, Nix et al.'s [25] protocol tends to amplify the most prevalent genome. For example, when further screened with enterovirus species specific primers, it was discovered that sample number 45 (Table 1) also had EVB88 in it (unpublished data). This was totally missed by Nix et al.'s [25] panenterovirus RT-snPCR screen. On the other hand, Nix et al.'s [25] panenterovirus RT-snPCR screen is not completely infallible. Failure on the part of the assay to amplify the gene of interest should not be considered with absolute certainty that the sample is negative for the virus of interest. For example, in another incident, Nix et al.'s [25] panenterovirus RT-snPCR screen failed to amplify the VP1 gene from an enterovirus isolate recovered on RD cell line in our laboratory. However, when species B and C specific RT-snPCR assays were used, echovirus 6 (E6) and poliovirus 1 (PV1) were detected, respectively. Hence, as valuable as this assay is, it also has its weaknesses. Consequently, strategies still have to be developed to improve its sensitivity as well as integrate it into already established enterovirus isolation protocols [26, 27]. In addition, Nix et al.'s [25] protocol consistently amplified an enterobacteriophage tail gene (Table 1) yielding a band that is similar in size to that expected for enteroviruses. Hence, the presence of a band in the expected range should be interpreted with caution pending the sequencing of the amplicon.

### 4.6. Conclusions

The results of this study showed the presence of CVA1, CVA3, CVA4, CVA8, CVA20, E32, EVA71, EVB80, and EVC99 in Ibadan, Southwestern Nigeria, in 2014. It thereby documents the first description of molecular sequence data on CVA1, CVA8, and E32 strains present in Nigeria. It further showed that species A enteroviruses were more commonly detected in the region when cell-culture bias is bypassed. The results of this study confirm that enteroviruses can be detected directly from faecal suspension using Nix et al.'s [25] protocol as proposed in the enterovirus surveillance guidelines [4]. Furthermore, the amplicons produced from Nix et al.'s [25] panenterovirus VP1 RT-snPCR assay are sufficient for sequencing and identification of the enteroviruses present in such samples. It further shows that Nix et al.'s [25] protocol tends to amplify the most prevalent genome when mixtures are present and failure on the part of the assay to amplify the gene of interest should not be considered with absolute certainty that the sample is negative for the virus of interest.

## Figures and Tables

**Table 1:** Samples positive for the enterovirus VP1 nested RT-PCR screen and the identity of enteroviruses detected in these samples.

S. number	Sample ID	Gender	Age (years)	VP1 RT-PCR	Serotype	Species
1	5	F	3	Positive	Unexploitable	
2	10	M	5	Positive	Unexploitable	
3	11	M	6	Positive	CVA1	Species C
4	15	M	2	Positive	CVA8	Species A
5	16	M	5	Positive	EVB80	Species B
6	20	M	2	Positive	CVA8	Species A
7	36	M	4.5	Positive	CVA20	Species C
8	41	M	1.5	Positive	EV71	Species A
9	43	F	1.5	Positive	EV71	Species A
10	44	M	1.5	Positive	Unexploitable	
11	45	M	1.5	Positive	CVA4	Species A
12	46	F	3.5	Positive	CVA3	Species A
13	48	F	4.5	Positive	Phage baseplate	
14	50	M	10	Positive	E32	Species B
15	59	M	10	Positive	EVC99	Species C

**Table:** (a) CVA20

Name	Length	Genus/species	Serotype, subgenogroup	Report	Genome
DQ358078.1∣CVA20_serotype_CAV2	7444	Enterovirus C	CVA20	Report	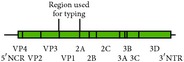

KF303091.1∣CVA20_NIG_2012	660	Enterovirus C	CVA20	Report	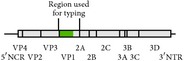

NIE2014_CV-A20_EV-C	354	Enterovirus C	CVA20	Report	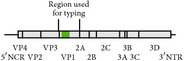

JX426682.1∣CVA20_T08-213	305	Enterovirus C	CVA20	Report	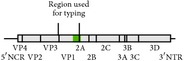

JX426681.1∣CVA20_T08-166	303	Enterovirus C	CVA20	Report	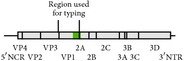

JX426680.1∣CVA20_T08-112	303	Enterovirus C	CVA20	Report	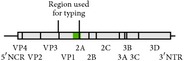

JX426679.1∣CVA20_MAR-252	303	Enterovirus C	CVA20	Report	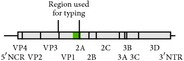

JX426678.1∣CVA20_MAR-250	305	Enterovirus C	CVA20	Report	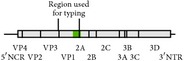

JX426677.1∣CVA20_MAR-249	305	Enterovirus C	CVA20	Report	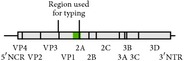

**Table:** (b) E32

Name	Length	Genus/species	Serotype, subgenogroup	Report	Genome
JQ411108.1∣E32_D614_M54-2009	259	Enterovirus B	E32	Report	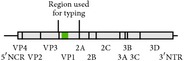

AF295475.1∣E32_50_99	568	Enterovirus B	E32	Report	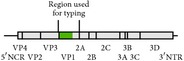

AF295518.1∣E32_PR_10	568	Enterovirus B	E32	Report	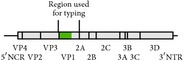

HQ662326.1∣E32_Mum-829	337	Enterovirus B	E32	Report	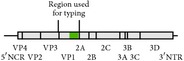

HQ662323.1∣E32_Mum-837	337	Enterovirus B	E32	Report	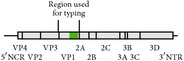

JN203970.1∣E32_N-990B	372	Enterovirus B	E32	Report	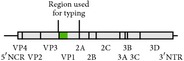

JN203969.1∣E32_N-900	375	Enterovirus B	E32	Report	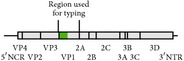

JN203968.1∣E32_N-737	876	Enterovirus B	E32	Report